# Anti-mosquito plants as an alternative or incremental method for malaria vector control among rural communities of Bagamoyo District, Tanzania

**DOI:** 10.1186/1746-4269-10-56

**Published:** 2014-07-11

**Authors:** Ester Innocent, Ahmed Hassanali, William NW Kisinza, Prince PP Mutalemwa, Stephen Magesa, Edmund Kayombo

**Affiliations:** 1 Institute of Traditional Medicine, Muhimbili University of Health and Allied Sciences, P.O. Box 65001, Dar es Salaam, Tanzania; 2 Department of Chemistry, School of Pure & Applied Sciences, Kenyatta University, P.O. Box 43844–00100, Nairobi, Kenya; 3 National Institute for Medical Research (NIMR), Amani Research Centre, P.O. Box 81, Muheza, Tanzania; 4 RTI International, Malaria Initiative, P.O. Box 1181–000621, Nairobi, Kenya

**Keywords:** Mosquitoes, Vector control, Ethno-knowledge, Medicinal plants, *Azadirachta indica*, *Annona* species

## Abstract

**Background:**

Plants represent one of the most accessible resources available for mosquito control by communities in Tanzania. However, no documented statistics exist for their contribution in the management of mosquitoes and other insects except through verbal and some publications. This study aimed at assessing communities’ knowledge, attitudes and practices of using plants as an alternative method for mosquito control among selected communities in a malaria-prone area in Tanzania.

**Methods:**

Questionnaires were administered to 202 respondents from four villages of Bagamoyo District, Pwani Region, in Tanzania followed by participatory rural appraisal with village health workers. Secondary data collection for plants mentioned by the communities was undertaken using different search engines such as googlescholar, PubMED and NAPRALERT.

**Results:**

Results showed about 40.3% of respondents used plants to manage insects, including mosquitoes. A broad profile of plants are used, including “mwarobaini” (*Azadirachta indica)* (22.5%), “mtopetope” (*Annona* spp) (20.8%), “mchungwa/mlimau” (*Citrus* spp) (8.3%), “mvumbashi/uvumbati” (*Ocimum* spp) (7.4%), “mkorosho” (*Anacadium occidentale*) (7.1%), “mwembe” (5.4%) (*Mangifera indica*), “mpera” (4.1%) (*Psidium* spp) and “maganda ya nazi” (4.1%) (*Cocos nucifera*). Majority of respondents collected these plants from the wild (54.2%), farms (28.9%) and/or home gardens (6%). The roles played by these plants in fighting mosquitoes is reflected by the majority that deploy them with or without bed-nets (p > 0.55) or insecticidal sprays (p >0.22). Most respondents were aware that mosquitoes transmit malaria (90.6%) while few respondents associated elephantiasis/hydrocele (46.5%) and yellow fever (24.3%) with mosquitoes. Most of the ethnobotanical uses mentioned by the communities were consistent with scientific information gathered from the literature, except for *Psidium guajava,* which is reported for the first time in insect control.

**Conclusion:**

This survey has indicated some knowledge gap among community members in managing mosquito vectors using plant. The communities need a basic health education and sensitization for effective exploitation of this valuable tool for reducing mosquitoes and associated disease burdens. On the other hand, the government of Tanzania should strengthen advocacy of botanical pesticides development, registration and regulation for public health benefits because they are source of pest control tools people rely on them.

## Background

Availability of healthcare services for improved diagnosis and treatment of mosquito-borne diseases have been considered as two crucial interventions in minimizing mortality and morbidity risk due to exposure to infected mosquitoes [1]. However, these alone cannot eliminate the high mosquito borne disease incidences in sub-Saharan Africa unless levels of infections and re-infections are substantially reduced through effective vector control mechanisms. Thus, a new strategy for control and prevention of mosquito-borne diseases, reinforcing linkages between health and environment and emphasizing Integrated Vector Management (IVM), has been advocated by WHO [1]. The strategy also stresses the importance of understanding the local vector ecology and local patterns of disease transmission. This is considered important in choosing the appropriate vector control tool from a range of the available options. IVM needs to be locally managed and flexible, with emphasis on decentralization, active community participation and harnessing of local knowledge [1]. In line with this understanding, Tanzania has since 2005 made a number of reforms in the health sector with most of the disease prevention and control program activities being planned and implemented at district level [2]. However, the current mosquito vector management efforts are focused on the use of Insecticide Treated Nets (ITN) and Indoor Residual Sprays (IRS). Very little effort has been directed towards the use of pesticidal plants that have been deployed by rural communities since time immemorial as a first line intervention in primary health care.

Furthermore, many of the synthetic insecticides available to-date faces challenges due to environmental contamination, resistance development by target insects and high deployment costs. This calls for the use of ecologically friendly and effective botanical insecticides as an alternative measure. The aim of present study, therefore, was to assess communities’ knowledge, attitudes and practices of using plants as an alternative or incremental method for mosquito vector control among selected communities in a malaria-prone area of Bagamoyo district, Tanzania so as to know the existing and potential contribution of anti-insect plants in this endeavor. Specifically, insights generated from this study, are expected to add value to the current IVM strategies in rural communities where plants continue to be deployed in mosquito control.

## Methods

### Study area and design

**Figure 1:**
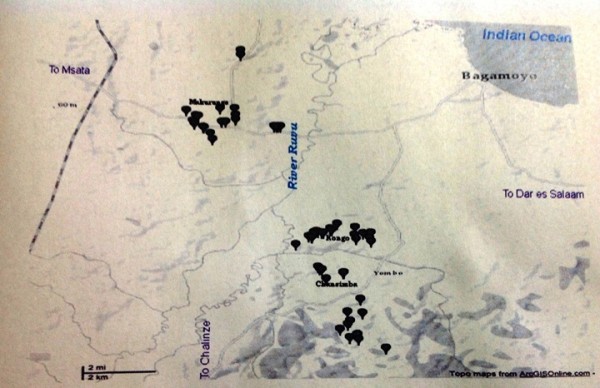
The map showing areas of concentrated water ponds in the four villages located along Ruvu River in Bagamoyo District.

Bagamoyo district is in Coast Region and it is allocated about 75 km north of Dar es Salaam which is the major city of Tanzania. The majority of the populations are ethnic groups of Wakwere, Wazaramo and Wazingua, however, other tribes co-exist in the area due to close proximity to Dar es Salaam. Majority of the population are Muslims; farmers and fishermen who practices Swahili Culture that was introduced by Arabs during the slavery and ivory trade in 19th century. The study was carried out in four villages of Yombo, Chansimba, Makurunge and Kongo (between 6° 24′ 19″ S: 38° 50′ 31″ E and 6° 29′ 03″ S: 38° 49′ 49″ E; Figure 1). The four villages are vicinity to river Ruvu which flows to the Indian Ocean. Over-flooding during the rainy seasons between April-May and October-November creates temporary and permanent mosquito breeding water ponds in the vicinity, which contribute to high infectivity rates during these periods. In addition, selection of the villages was based on previously reported higher entomological inoculation rates [3] data, and malaria epidemiology, demography and entomological data of the study area [4-7]. The study was based on a cross-sectional design using self-administered questionnaires conducted in the selected local communities. Consented respondents (202) were randomly selected on the criteria that they were at least in the post-primary school age (i.e. 14 years and above).

### Data collection

Before commencement of the study, reconnaissance surveys were conducted in the study area. The purpose of the visits was to get acquainted with the study areas and discuss the project with district and village leaders. Leaders in all the four villages agreed to convene a meeting of the adult residents with the help of Village Health Workers (VHWs) who identified the participants based on our criteria. Informed collective agreement was made and other useful information was sought. Those who consented proceeded to filling in the self-administered questionnaires and then followed by in-depth interview in a focus group discussion. During filling of questionnaires those who did not know how to read and write were assisted to fill the questionnaires by the research team who asked them to respond to questions. Focus group discussion consisted of leaders of the village and village health workers whom together checked and confirm identity of the meaning and of any ambiguities. However, FGD did not affect the priority of list which was given by individuals when filling the questions. Semi-structured questionnaire and focus group discussions were translated in Swahili language (after being translated from the Standard English version). Published information on the plant species mentioned was gathered through literature search using google scholar, PubMED and NAPRALERT. The final results of the study were shared with all study participants during community feedback meetings at each of the study sites.

### Ethical considerations

Scientific and ethical approval for the study (NIMR/HQ/R.8a/Vol. IX/1093) was granted by the Medical Research Coordinating Committee (MRCC) of the National Institute for Medical Research and the institutional clearance was granted by the Senate, Research and Publication of the Muhimbili University of Health and Allied Sciences (MUHAS/RP/AEC/VOL.XIII/), Tanzania.

### Data analysis

Quantitative data were double entered into a computer database using *EpiData® software.* Responses from closed*-*ended questions in questionnaires were coded before being entered into the computer. Data quality checking process was done and thereafter the database was exported to STATA 10 software for statistical analysis.

## Results

### Socio-economic and demographic profile

Respondents (202) were interviewed from four villages, Yombo (48), Chasimba (55), Kongo (49) and Makurunge (50). Majority of the respondents were of age ranging between 26–39 years (54.5%) and were married (60.2%). There was, however, no significant difference between the sex categories of the respondents (p > 0.32). Majority of the respondents had primary school level of education (66.7%) and were peasants (58.2%) who had stayed in the study villages for varying periods (>15 years, 38.1%; 11–15 years, 17.5%; 6–10 years, 16.5%; 2–5 years, 15.5%; <1 year, 12%). From the questionnaires and on general inspection of their homesteads, majority were of mud and metal roof (50.3%) or mud and grass roof (30.4%); the rest were of blocks with metal roof (18.4%) and few with blocks and grass or blocks and tiles (Table 1).

**Table 1:** Respondents’ socio-economic and demographic profile (N = 202)

**Variable**	**Total (N = 202)**	**Number/proportion (%) of respondents**				***χ*** **2 (p-value)**
		**Yombo**	**Chasimba**	**Kongo**	**Makurunge**	
***Sex***						
Male	106 (52.5)	28 (58.3)	25 (45.5)	23 (46.9)	30 (60.0)	3.49 (0.32)
Female	96 (47.5)	20 (41.7)	30 (54.6)	26 (53.1)	20 (40.0)	
***Age group***						
< 15	8 (3.96)	0 (0.0)	4 (7.27)	1 (2.04)	3 (6.0)	21.41 (0.05)
15-25	8 (16.67)	16 (29.09)	10 (20.41)	12 (24)	46 (22.8)	
26-39	110 (54.5)	22 (45.8)	31 (56.4)	29 (59.2)	28 (56.0)	
40-55	31 (15.4)	14 (29.2)	3 (5.5)	8 (16.3)	(12.0)	
> 55	7 (3.5)	4 (8.3)	1 (1.8)	1 (2.0)	1 (2.0)	
***Marital status***						
Married	118 (60.2)	31 (67.4)	23 (42.6)	31 (67.4)	33 (66.0)	22.54 (0.03)
Single	61 (31.1)	7 (15.2)	26 (48.2)	12 (26.1)	16 (32.0)	
Widow	6 (3.1)	3 (6.5)	2 (3.7)	1 (2.2)	0 (0.0)	
Divorced	5 (2.6)	2 (4.4)	1 (1.9)	2 (4.4)	0 (0.0)	
Cohabiting	6 (3.1)	3 (6.5)	2 (3.7)	0 (0.0)	1 (2.0)	
***Education level***						
No formal education	22 (11.0)	7 (14.6)	3 (5.6)	7 (14.3)	5 (10.0)	21.40 (0.05)
Primary school	134 (66.7)	35 (72.9)	29 (53.7)	35 (71.4)	35 (70.0)	
Form IV	38 (18.9)	5 (10.4)	18 (33.3)	5 (10.2)	10 (20.0)	
High school	4 (2.0)	0 (0.0)	2 (3.7)	2 (4.1)	0 (0.0)	
Higher education	3 (1.5)	1 (2.1)	2 (3.7)	0 (0.0)	0 (0.0)	
***Time of residential stay***						
<= 1 year	24 (12.4)	5 (10.9)	9 (17.3)	8 (17.0)	2 (4.1)	
2 - 5 yrs	30 (15.5)	4 (8.7)	7 (13.5)	8 (17.0)	11 (22.5)	
6 - 10 yrs	32 (16.5)	4 (8.7)	10 (19.2)	10 (21.3)	8 (16.3)	15.40 (0.22)
11 - 15 years	34 (17.5)	9 (19.6)	10 (19.2)	5 (10.6)	10 (20.4)	
> 15 yrs	74 (38.1)	24 (52.2)	16 (30.8)	16 (34.0)	18 (36.7)	
***Types of homesteads***						
Mud and grass	61 (30.4)	16 (34.0)	12 (21.8)	15 (30.6)	18 (36.0)	
Mud wall and metal roof	101 (50.3)	26 (55.3)	25 (45.5)	28 (57.1)	22 (44.0)	
Block wall and grass roof	1 (0.5)	0 (0.0)	0 (0.0)	0 (0.0)	1 (2.0)	19.34 (0.08)
Block wall and metal roof	37 (18.4)	4 (8.5)	18 (32.7)	6 (12.2)	9 (18.0)	
Block wall and tiles	1 (0.5)	1 (2.1)	0 (0.0)	0 (0.0)	0 (0.0)	
***Occupation***						
Peasant	117 (58.2)	34 (70.8)	24 (44.4)	28 (57.1)	31 (62.0)	28.3 (0.005)
Housewife	24 (11.9)	4 (8.3)	5 (9.3)	10 (20.4)	5 (10.0)	
Self employed	28 (13.9)	4 (8.3)	10 (18.5)	5 (10.2)	9 (18.0)	
Civil servant	27 (13.4)	4 (8.3)	15 (27.8)	6 (12.2)	2 (4.0)	
Casual employment	5 (2.5)	2 (4.2)	0 (0.0)	0 (0.0)	3 (6.0)	

### People’s knowledge and practice on use of plants for controlling mosquitoes

About 81 (40.3%) of the respondents reported to have used plants to control arthropods including mosquitoes (30.3%) and scorpions (11.1%). Others were spiders, centipedes, army ants, bedbug, cockroaches, bees, termites, small ants, house flies, etc. Snakes which do not belong to the phylum arthropoda, was ranked second in terms of organisms that attack human habitats and it is also controlled with plant products. Most of the arthropods mentioned were vectors of important tropical diseases or cause other harm to humans (Table 2).

**Table 2:** List of the most invasive insects/organisms controlled by plants

**Sno.**	**Swahili name**	**Common english name**	**Order**	**No. of responses (%)**
1	Mbu	Mosquito	Diptera	30 (30.3)
2	Nyoka	Snake	Squamata	15 (15.3)
3	Ng’e	Scorpion	Scorpiones	11 (11.1)
4	Buibui	Spider	Argiope	10 (10.1)
5	Siafu	Army ant	Hymenoptera	6 (6.1)
6	Kunguni	Bed bug	Hemiptera	6 (6.1)
7	Tandu	Centipede	Scolopendromorpha	6 (6.1)
8	Mende	Cockroach	Blattaria	4 (4.0)
9	Nyuki	Bee	Hymenoptera	4 (4.0)
10	Mchwa	Termite	Blattaria	3 (3.0)
11	Others			4 (4.0)

Further probing on the profiles of plants used in managing the arthropods identified *Azadirachta indica*, *Annona* spp*, Ocimum* spp*., Citrus* spp*., Anacardium occidentale Mangifera indica, Psidium* spp and *Cocos nucifera* as the plant species commonly used (Table 3). The distances travelled by members of the community to collect the plants were less than one kilometer for majority of respondents (51.2%), while only 6.1% travelled up to 5 km. Most of the plants are collected from the wild (54.2%), with some from respondents’ farms (28.9%) and home gardens (6%).

**Table 3:** Plants used for insect control in Bagamoyo District

**S. No**	**Swahili name**	**Species/Genus name (Voucher specimen number)**	**Family**	No. of responses (%)*
1	Mwarobaini, mwarobaini kamili	*Azadirachta indica* (ITM 3080)	Meliaceae	38 (22.5)
2	Mtopetope, mtopetope mwitu, mtomoko, mtomoko mwitu, mchekwa, mtopetope pori	*Annona squamosa* (ASS-T-II)*, An. senegalesis* (OT 00353)	Annonaceae	29 (17.2)
3	Mchungwa, limau	*Citrus limonium* (ITM 433)	Rutaceae	14 (8.3)
4	Mvumbashi, uvumbati	*Ocimum suave* (ITM 445.0303)	Laminaceae	13 (7.7)
5	Mkorosho	*Anacardium occidentale* (TMRU 2876)	Anacardiaceae	12 (7.1)
6	Mwembe	*Mangifera indica* (TMRU 963)	Myrtaceae	10 (5.4)
7	Mpera	*Psidium guajava* (TMRU 2880)	Myrtaceae	7 (4.1)
8	Maganda ya nazi	*Cocos nucifera* (TMRU 1510)	Arecaceae	7 (4.1)
10	Mstafeli,	*An. muricata* (OT 00351)	Annonaceae	6 (3.6)
11		Others	-	34 (22.1)

*Multiple responses were allowed.

Respondents who acknowledged using plants in controlling mosquitoes had different modalities and time for usage. Most of them either use plants daily (56.3%) or when need arises (25.0%). Parts of the plants frequently mentioned for use include leaves (38.2%) and roots (41.2%). These are put on burning charcoal in containers placed at different locations inside the homesteads to generate smoke and volatile emissions (45.2%). According to the majority of the respondents (78.2%), the process between application of the plant products and insects dying took less than six hours (Table 4). Other modalities of application of plant parts were placing ground fresh materials (21.9%) or small pieces (12.5%) at selected places within the homesteads, and soaking plant parts or powder in water and then spraying (15.6%). Majority of the respondents appeared to target adult mosquitoes in their control efforts because most plant products were applied inside homesteads (45.2%) rather than at dumping areas (19.4%), water tanks (9.7%) or outdoor sewage systems (3.2%).

**Table 4:** Knowledge and practice of using plants in mosquito controls among Bagamoyo communities

**Variable**	**No. of respondents**	**%**
***How long does it take for the insect to die?***		
less than 1 hour	11	34.38
1-6 hours	14	43.75
7-12 hours	6	18.75
13-24 hours	1	3.13
***How frequent do you apply***		
once a day	18	56.3
once a week	2	6.3
once a month	2	6.3
once a year	2	6.3
once necessary	8	25.0
***Modality of using/applying the plant***		
Cut to pieces and distribution	7	21.9
Ground fresh materials distribution	4	12.5
Soaking and spraying	5	15.6
Smoking	14	43.8
Placed in a ceiling	2	6.3
***Distances traveled to harvest the plant***		
< 1 km	42	51.2
1-2 km	17	20.7
2-5 km	6	7.3
5 km		
***Where do you apply it***	***No***	***%***
Inside the house	14	45.2
in dumping areas	6	19.4
Around the house premise	7	22.6
In water tanks	3	9.7
In sewage systems	1	3.2
**Part of the plant used**	**No**	**%**
Stem	1	2.9
Leaves	13	38.2
Roots	14	41.2
Fruits	5	14.7
Seeds	1	2.9
***Place of harvesting the plant***	***No***	***%***
Farm	24	28.9
Home garden	5	6.0
Roadside	2	2.4
Wild	45	54.2
Forest reserve	7	8.4

### Knowledge about mosquito transmitted diseases, multiplication and control

Majority of respondents (97.8%) were aware of disease agents transmitted by mosquitoes. Among the diseases mentioned included malaria (90.6%), elephantiasis/hydrocele (46.5%) and yellow fever (24.3%) (Table 5). Furthermore, a large proportion of respondents associated mosquito breeding and multiplication with stagnant water (70.8%), dumping sites (35.1%), sewages (45.5%) and drainage systems (26.2%). Others included uncleared bushes around the houses (35.6%) and leaking taps (13.4%). Further probing with respondents on mosquito preventive measures, identified bed-nets (63.4%) and drying stagnant water bodies (59.9%) as the best options, in addition to keeping home premises clean (34.2%), using insecticides residual sprays (32.2%) and using plants/herbs (17.3%) (Table 5). However, majority of the respondents were not comfortable with reliance on only one preventive measure such as ITN, or use of plants/herbs or insecticide residual spray because responses showed no significant differences between the uses of the three methods in all the four villages surveyed (Table 6).

**Table 5:** Knowledge of mosquito transmitted diseases, multiplication and control (N = 202)

**Variable**	No*	**%**
***Knowledge of diseases caused by mosquitoes***		
*Elephantiasis*	*118*	*58.4*
*Hydrocele*	*70*	*34.7*
*Malaria*	*183*	*90.6*
*Yellow fever*	*49*	*24.3*
*HIV*	*14*	*6.9*
*All of the above*	*1*	*0.5*
***Knowledge of places of mosquito breeding***		
Water and air	22	10.9
Water and bush	72	35.6
Stagnant water alone	143	70.8
Air alone	9	4.5
Bush alone	43	21.3
Dumping sites	71	35.1
Sewage systems	92	45.5
Drainage systems	53	26.2
Leaking taps	27	13.4
***Preventive measures***		
Using bednet	128	63.4
Using treated bednet	75	37.1
Using plants/herbs	35	17.3
Filling stagnant water bodies	121	59.9
Using insecticides residual sprays	65	32.2
Keeping home premises clean	69	34.2
Inspecting water bodies around the house premises	46	22.8
Wearing long sleeves	19	9.4
All of the above	15	7.4
None of the above	4	2.0

^*^Multiple responses were allowed.

**Table 6:** Respondents’ reliance on various mosquito preventive measures

	**Village**				**Total**	***χ*** **2 (p-value)**
	**Yombo**	**Chasimba**	**Kongo**	**Makurunge**		
***Use of ITN***						
Yes	14 (30.4)	23 (42.6)	19 (41.3)	21 (42.9)	77 (39.5)	2.1 (0.55)
No	32 (69.6)	31 (57.4)	27 (58.7)	28 (57.1)	118 (60.5)	
***Use of plants/herbs***						
Yes	3 (6.4)	10 (18.5)	9 (18.8)	13 (26.5)	35 (17.7)	6.82 (0.08)
No	44 (93.6)	44 (81.5)	39 (81.3)	36 (73.5)	163 (82.3)	
***Insecticide residual spray***						
Yes	10 (21.3)	19 (35.2)	16 (33.3)	20 (40.8)	65 (32.8)	4.4 (0.22)
No	37 (78.7)	35 (64.8)	32 (66.7)	29 (59.3)	133 (67.2)	

### Perceptions on the use of plants in mosquito control

On respondents’ attitude towards incorporating plants in mosquito management, their accessibility (26.7%) and affordablility (29.2%) were important factors (Table 7). The other reason highlighted was that the use of plants has been an old and familiar traditional practice (22.8%). However, the respondents were willing and happy to participate in other community-based mosquito management practices, such as draining off or reducing formation of small stagnant water bodies around their houses (58.4%), cleaning bushes (35.6%) and applying safe insecticides on sizeable stagnant waters (34.2%) (Table 7).

**Table 7:** Attitude about elimination of mosquitoes (N = 202)

**Variable**	^***#***^**No of response**	**%**
***Attitude towards eliminating mosquitoes from our homestead***		
No, because mosquitoes are created by God	17	8.4
Mosquitoes come with rain no one can control them	35	17.3
No, mosquitoes are only seen after sun set	14	6.9
Yes, by eliminating stagnant water	81	40.1
Yes, by closing widows and doors	22	10.9
Yes, by using indoor insecticide residual spray	65	32.2
Yes, by spraying insecticide in stagnant water	91	45.0
Yes, by using ITN	77	38.1
***Participation in mosquito control***		
Destroying or avoid creating stagnant water bodies	118	58.4
It is the responsibility of the government	9	4.5
Wait for the directives from the district malaria control focal person	8	4.0
Community based programs of cleaning bushes	72	35.6
Community based programs of applying safe insecticides in stagnant waters	69	34.2
***Reliance of plants as source of insecticides***		
We use them often	35	17.3
It is an old practice	46	22.8
We have many plants around us	54	26.7
Not harmful like insecticides bought in the shop	40	19.8
Plants are affordable, unlike insecticides	59	29.2

^#^*Multiple responses were included.*

### Secondary data generated on ethnobotanical status and scientific investigations on the plants mentioned

Secondary data collection on plants mentioned by the communities in Bagamoyo district was done by using different search engines such as Google Scholar, PubMED and NAPRALERT. Except for *Psidium* species (the use of which is reported for the first time), all plants mentioned have been either reported to be used traditionally elsewhere to control some insects or investigated scientifically and evidence on the presence of anti-insect phytochemicals generated (Table 8). Interestingly, control methods deployed in the ethnobotanical practices, as well as scientific investigations undertaken, both targeted repellency and/or larvidical properties, similar to the use of the plants by the communities in Bagamoyo district (Tables 4 and 8).

**Table 8:** An overview of Insecticidal plant efficacy from literature review of selected species mentioned in the Bagamoyo survey

	**Swahili name**	**Species/genus name**	**Name of other related species growing in Tanzania**	**Related Ethno botanical uses in insect management**	**Scientific studies**
1	Mwarobaini	*Azadirachta indica* (Maliaceae)		In Tanzania, leaves mixture with cow urine controls maize pests in the field; Also, infusion of leaves and tobacco powder are sprayed to control crop pests in the field [8]	Dried leaf powder is used to repel *Culex quinquefasciatus*[9].
					Larvicidal activity against *Aedes aegyptis*[10].
				Leaf, seed, seed oil, flower and fruit are used by Indians for control of Rice weevil [11].	
2	Mtopetope;	*Annona squamosa*	*Annona cherimoya*	Indians use leaf, bark, root, stem and fruits for control of head lice and insects [11].	Leaf extract of *A. senegalensis and A. squamosa* is used against mosquito larvae [12,13].
	Mtopetope		*Annona reticulata* L.		
	mwitu;		*Annona stenophylla*		
	Mtomoko	*Annona senegalesis* (*Annonaceae*)	Engl. [14,15].	*Annona senegalensis* Pers is used traditionally in Nigeria to treat victims of snakebite [16].	Also leaf extract is used against *Aedes adopticus*[17].
	Mchekwa;		*Annona Montana*[18].		
	Mtopetope pori				
					*Annona senegalensis*
					leaves was effective against different stages of *Caryedon serratus*
					development [19].
3	Mchugwa; Limau	*Citrus spp* (*Rutaceae*)	*C. aurantifolia*	Dried leaf of *C. limonium* is used against wheat weevil and flour beetle by Indians [11,20].	Essential oils of *C. aurantifolia*, *C. paradis, C. sinensis and C. limonium* is used for control of Cowpea weevils (*Callosobruchus maculatus*) [21].
			*C. paradis*		
			*C. sinensis*		
			*C. limonium*		
			*C. aurantium*		
			*C. reticulate* Blanco [14,15].		*C. aurantium*. Essential oils are used to control tomato moth (*Tuta absoluta*) and Africa cotton leaf worm. (*Spodoptera littoralis*) [22].
					Show bioefficacy against *Ae. albopictus* of three Citrus essential oils, derived from peels of *Citrus sinensis, Citrus limon,* and *Citrus paradise* and of their component [23]; Also against *An. gambiae*[24].
4	Mvumbashi	*Ocimum Spp* (*Laminaceae*)	*O. americanum*	Leaves of *O. suave* are arranged in a bag of millet or maize to control pests [8].	Essential oils of *O. suave* and *O. kilimandscharium* are *Cx. Quinquefasciatus* and *Anopheles arabiensis* repellant [25].
	Uvumbati		*O. suave*		
			*O. lamiifolium*		
			*O. polystachyon*	Leaves of *O. gratissimum* are used in Nigeria in post harvest protection of maize [26].	
			*O. grantissimum*		Essential oils of *O. canum* and *O. basilicum* are used for control of Cowpea weevils (*Callosobruchus maculatus*) [27].
			*O. kilimandscharium*		
			*O. canum*		
			*Hyptis suaveolens* (Formally, *O. basilicanum*)		High protection time of essential oil of *O. basilicum* with ethyl alcohol, tested against three mosquito species, *Aedes aegypti, Anopheles minimus and Culex quinquefasciatus*[28].
			[14,15].		
			*O. albosteblatum*		
			*O. angustifolium*		
					*O. gratissimum* essential oil formulation repelled anopheline and culicine mosquitoes [29].
			*O. obovatum*[14].		
					*O. basilicum* essential oil showed the strongest larvicidal activity while *O. gratissimum* exhibited the longest duration of action for mosquito repellent activity [30].
5	Mkorosho	*Anacardium occidentale* (*Anacardiaceae*)		The gum from stem of *A. occidentale* is used as an adhesive (for woodwork panels, plywood, bookbinding), partly because it has insecticidal properties [31].	Powders and extracts of *A. occidentale* seeds were effective in controlling cowpea bruchid, *C. maculatus* in stored cowpea seeds [32].
					Larvicidal activities of aqueous extracts of Leaf, Bark and Nutshell of *A. occidentale* showed insecticidal properties on the *An. gambiae*[33].
6	Mstafeli	*An.muricata*		Leaves of *An. muricata* are used by phu thai tribe in Lao People’s Democratic Republic to repel bedbugs and lice [20].	*Annona muricata* shows promising larvicidal activity against *Ae. Eagypti*[13,34].
7	Mwembe	*Mangifera indica*		*Leaves of M. indica* is used in uMkhanyakude district, KwaZulu-Natal province, South Africa as mosquito insect repellent [35].	
		(*Myrtaceae*)			
8	Maganda ya nazi	*Cocos nucifera*			Coconuts oil is used as mosquito and tick repellant [36,37].
		(*Arecaceae*)			
9	Mpera	*Psidium Spp*	*P. guajava*		
		(*Myrtaceae*)	*P. cattleianum*		
			*P. friedrichsthalianum*		
			*P. guineese* ([14,15]).		

## Discussion

The Government of Tanzania has invested in a number of interventions aimed at alleviating mosquito-borne diseases such as malaria and lymphatic filariasis. These include improving diagnosis and treatment of the diseases, provision of subsidized anti-malarial (ALU) drugs, and use of insecticide-treated nets (ITN). Elsewhere, history and scientific evidence show that the battle against mosquito-borne diseases has succeeded significantly through massive spraying with DDT [38,39], although ecological unfriendliness of the insecticide has made its continued use very controversial. However, this may have opened up the use of plant natural products with subtle anti-insect effects as a better alternative in reducing the burden of mosquito-borne diseases. Specific tropical plants are readily accessible by rural communities, and are eco-friendly and cost- effective.

Although only 40.3% of the respondents in the Bagamoyo District reported using plants in the control of insects and especially mosquitoes. Many of the mentioned plants are exotic, although they were introduced on the African coastal area long time ago, and are from plant families with anti-insect activities [40-42]. Of special significance is that the majority of respondents were open to the possibility of using a combination of different methods in an integrated vector management and were aware of different diseases caused by mosquito species such as malaria, elephantiasis/hydrocele and yellow fever. This could be attributed to regular community-based sensitizations from other malaria interventions such as the Bagamoyo Bednet [3,43,44] and on-going Malaria Vaccine Trial [45]. The present study identified the need for regular outreach education on proper deployment of anti-insect plants within rural communities where there is continued use of this natural resource to add value to the current mosquito and malaria control strategies. Further R&D on the plant products deployed, their efficacy and modes of action would lay down the groundwork for selecting those that are particularly effective in different uses and in optimizing their deployment.

## Conclusion

The present survey indicates that a good proportion of members of different communities in Bagamoyo District continue to use plants to control different disease vectors and other pests and that the majority are open to the possibility of integrating them with other interventions. Continued use of these medicinal plants needs to be encouraged and promoted as they have potential for complementing other interventions in vector and disease control. Tanzania has no clear policy or guidelines on development, registration and use of botanical insecticides. Elsewhere, some botanical insecticides have been developed for multipurpose uses in pest control, including mosquito control. This calls for the government to strengthen advocacy of botanical pesticides development, registration and regulation for public health benefits.

## Competing interests

The authors declare that they have no competing interests.

## Authors’ contributions

IE, MSM and HA contributed to the study conception, design, fieldwork, data analysis, interpretation and drafting, revision and final approval of the manuscript. KNW, MPP and KE contributed to fieldwork, data analysis, data interpretation, revision and final approval of the manuscript. All authors read and approved the final manuscript.
